# Structural Insights Into the Dynamic Evolution of Neuronal Networks as Synaptic Density Decreases

**DOI:** 10.3389/fnins.2019.00892

**Published:** 2019-08-22

**Authors:** Ye Yuan, Jian Liu, Peng Zhao, Fu Xing, Hong Huo, Tao Fang

**Affiliations:** ^1^Department of Automation, Shanghai Jiao Tong University, Shanghai, China; ^2^Key Laboratory of System Control and Information Processing, Ministry of Education, Shanghai, China

**Keywords:** evolving network model, synaptic density, network efficiency, network connectivity, scale-free network

## Abstract

The human brain is thought to be an extremely complex but efficient computing engine, processing vast amounts of information from a changing world. The decline in the synaptic density of neuronal networks is one of the most important characteristics of brain development, which is closely related to synaptic pruning, synaptic growth, synaptic plasticity, and energy metabolism. However, because of technical limitations in observing large-scale neuronal networks dynamically connected through synapses, how neuronal networks are organized and evolve as their synaptic density declines remains unclear. Here, by establishing a biologically reasonable neuronal network model, we show that despite a decline in the synaptic density, the connectivity, and efficiency of neuronal networks can be improved. Importantly, by analyzing the degree distribution, we also find that both the scale-free characteristic of neuronal networks and the emergence of hub neurons rely on the spatial distance between neurons. These findings may promote our understanding of neuronal networks in the brain and have guiding significance for the design of neuronal network models.

## Introduction

The human brain contains hundreds of millions of neurons, which form structurally complex and computationally efficient networks through dynamic synapses (Laughlin and Sejnowski, 2003; Bassett and Sporns, 2017). To ultimately gain insight into the design principles of neuronal networks, a necessary and fundamental step is to investigate the structure of neuronal networks in the brain. Unfortunately, because the number of neurons and synapses is too large, understanding how neuronal networks organize and evolve is still one of the enduring challenges of modern neuroscience.

Over the past decades, much important progress has been made in studying neuronal networks in the brain, and some organizing mechanisms in neuronal networks have been revealed at the mesoscopic level. Spike timing-dependent plasticity (STDP), one of the most widely studied synaptic characteristics, has been demonstrated in various neuronal systems over a wide spectrum of species, from insects to humans (Caporale and Dan, 2008). Because of synaptic plasticity, neuronal electrical activities can not only encode information but also contribute to the structural refinement of neuronal networks. Further studies on the cellular mechanisms of synaptic plasticity indicate that the formation, change, and elimination of synaptic connections may be mediated by neuromodulators and glia (Picciotto et al., 2012; De Pitta et al., 2016). In particular, glia are involved in almost all aspects of the development of neuronal networks in the brain (Cody and Freeman, 2013). In addition, the structure of neuronal networks must evolve toward optimal energy efficiency as a consequence of the limited total metabolic energy in the brain. Metabolic energy, as a unifying principle governing neuronal biophysics, has been demonstrated in many experimental and theoretical studies (Niven and Laughlin, 2008; Hasenstaub et al., 2010). Furthermore, studies show that the neuronal network construction in the brain should go through the following two stages: synapses proliferate far more than normally required in the early stage, and then overproduced synapses are selectively pruned until the synaptic density (i.e., the ratio of the number of synapses to that of neurons) is almost stable (Paolicelli et al., 2011; Navlakha et al., 2015). Such neuronal network construction in the brain can then greatly reduce the large amounts of genetic information used to encode its structure (Thompson et al., 2001; Glahn et al., 2010).

During the evolution, changes in the structure of neuronal networks are affected by many factors. There is much evidence that the absence of any one or some of these factors may induce the structure of neuronal networks to evolve toward undesirable, even disordered directions (Holmes et al., 2019), such as autism (Amaral et al., 2008), Alzheimer's disease (He et al., 2008), and Parkinson's disease (Helmich et al., 2010). Although some biological mechanisms during evolution have been revealed, limited by current techniques and methods in neuroscience, it is difficult to observe and record neural activities and their structural changes of large-scale neuronal networks at the mesoscopic level. There are still many challenging issues to be addressed, such as how to judge which synapses should be pruned, how synapses are formed, how neuronal networks evolve toward optimal energy efficiency, and how hub neurons in neuronal networks appear. Nonetheless, there is a potential way to study the structural changes of neuronal networks by integrating these factors into a computational model.

Here, a neuronal network model is developed to simulate the dynamic structural evolution process of neuronal networks. In this model, the change in synaptic weights follows a synaptic learning rule, and the formation and elimination of synaptic connections can occur at the same time. In the following, we use this model to (1) study the change of network connectivity during the decline of the synaptic density, (2) reveal the relationship between network efficiency and the decline of the synaptic density, and (3) investigate the degree distribution of the neuronal network model. Finally, we will discuss the simulation results of this paper in combination with previous studies.

## Methods

### Model Construction

Network structure generally serves as a critical driver for complicated functions in a broad class of systems across many research fields, such as gene regulatory networks (Hasty et al., 2001), biological neuronal networks (Bullmore and Sporns, 2009), artificial neuronal networks (Xu et al., 2019), and social networks (Borgatti et al., 2009). Consider a neuronal network of *N* neurons, in which the leaky integrate and fire model is used to simulate the dynamics of the neuron membrane potential as follows (Dayan and Abbott, 2001; Izhikevich, 2004):

(1) $$\begin{array}{l} \frac{dx_{j}\left(t\right)}{dt}=-\frac{1}{\tau_{m}}\cdot x_{j}\left(t\right)+\frac{R}{\tau_{m}}\cdot \overset{N}{\underset{i=1,i\neq j}{\sum }}w_{ij}\cdot \varepsilon_{i}\left(t,t_{i}\right)\cdot e_{ij}\\ \;+\frac{R}{\tau_{m}}\cdot \overset{M}{\underset{k=1}{\sum }}b_{kj}\cdot u_{k}\left(t,t_{k}\right)\cdot e_{kj},\;\left(j=1,2,\ldots ,N\right)\\ \;if\;x_{j}\left(t\right)>x_{th},\;then\;x_{j}\left(t\right)\leftarrow 0 \end{array}$$

(2) $$\begin{array}{r} \varepsilon_{i}\left(t,t_{i}\right)=\rho \cdot \frac{t-t_{i}}{\tau_{sys}}\cdot e^{-\frac{t-t_{i}}{\tau_{sys}}},t\geq t_{i} \end{array}$$

(3) $$\begin{array}{r} u_{k}\left(t,t_{k}\right)=\rho \cdot \frac{t-t_{k}}{\tau_{sys}}\cdot e^{-\frac{t-t_{k}}{\tau_{sys}}},t\geq t_{k} \end{array}$$

where *x*_*j*_(*t*) denotes the membrane potential of the *j-th* neuron at time *t*. ε_*i*_(*t, t*_*i*_) denotes the output signal of the *i-th* neuron at time *t*, and *w*_*ij*_ (0 ≤ *w*_*ij*_ ≤ 1) denotes the weight of the synaptic connection from the *i-th* to the *j-th* neuron. *u*_*k*_(*t, t*_*k*_) denotes the output signal produced by the *k-th* external signal source at time *t*, and *b*_*kj*_ (0 ≤ *b*_*kj*_ ≤ 1) is the weight of the synaptic connection from the *k-th* external signal source to the *j-th* neuron. The value of *e*_*ij*_ and *e*_*kj*_ can only be 1 or 0, indicating the presence or absence of the corresponding connection, respectively. *M* denotes the number of external signal sources. Like neurons, the external signal sources generate spikes at a given frequency, whose output signals can be calculated from Equation (3) according to the firing time of their latest spikes. *R* denotes the resistance coefficient, and τ_*m*_ is the rate of change of the membrane potential. *x*_*th*_ is the threshold of the membrane potential. *t*_*i*_ and *t*_*k*_ denote the firing time of the latest spikes of the *i-th* neuron and the *k-th* external signal source, respectively. τ_*sys*_ is a time constant, and ρ is a gain coefficient. Obviously, one neuron can receive signals from other neurons within this network simultaneously, as well as from signals outside this network, and its membrane potential can change over time. The default values of the above parameters are *R* = 1.0 × 10^6^ Ω, τ_*m*_ = 100 ms, *x*_*th*_ = 85.0 mV, τ_*sys*_ = 20 ms, and ρ = 8.0 × 10^−7^.

**Figure 1:**
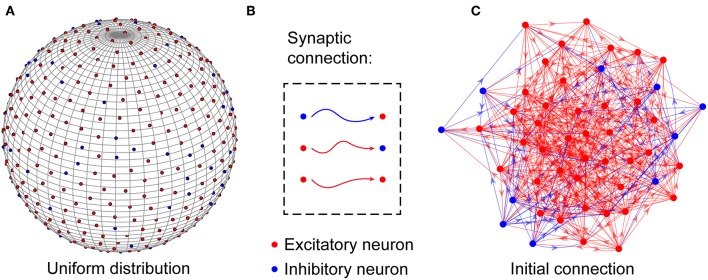
Structural organization of the neuronal network. **(A)** Excitatory and inhibitory neurons are placed uniformly on the surface of a unit sphere. **(B)** In the network, excitatory neurons can be connected to any neurons, but inhibitory neurons can only be connected to excitatory neurons. **(C)** Before the evolution of the network, the neurons are randomly connected to each other to build a large number of connection paths, which can be selectively pruned during the subsequent evolution. Obviously, the neuronal network is directed. For simplicity, only a portion of the neurons and synaptic connections are drawn in the figure.

At the microcircuit level, numerous studies have shown that neuronal networks in the brain are not organized randomly (Song et al., 2005); instead, they follow a distance-dependent connection pattern (Ercsey-Ravasz et al., 2013; Wang and Kennedy, 2016; Wiles et al., 2017). To reflect this distance dependence, neurons in the neuronal network are uniformly placed on the surface of a unit sphere, as shown in Figure 1A and are connected to each other based on a probability function of distance-dependent connection defined later. According to experimental findings (Markram et al., 2004), the neuronal network is assumed to contain *N*^*E*^ excitatory and *N*^*I*^ inhibitory neurons, i.e., *N* = *N*^*E*^ + *N*^*I*^, and their ratio is set to 4:1. For simplicity, inhibitory neurons are used to balance the activity of the entire network (Hattori et al., 2017), and the synaptic weights between excitatory neurons and inhibitory neurons are assumed to remain fixed. In the neuronal network, excitatory neurons can be connected to any other neurons, but inhibitory neurons can only be connected to excitatory neurons (Salinas and Sejnowski, 2000). That is, there are three types of synaptic connections in the neuronal network: those from excitatory to excitatory neurons, those from excitatory to inhibitory neurons, and those from inhibitory to excitatory neurons, as shown in Figure 1B. Note that signal transmission via synaptic connections is unidirectional, but the existence of synaptic connections with opposite directions between two neurons is also allowed in the neuronal network. Here, to accord with the developmental characteristics of neuronal networks in the brain, the neurons are randomly but densely connected to each other, as shown in Figure 1C. During network evolution, unimportant synapses can be selectively pruned to optimize the network structure according to the evolution rule proposed later.

### Synaptic Learning Rule

Neurons in the brain interact with each other through synaptic connections, and brain functions such as the formation of memories and the learning of actions, are closely related to the changes of synaptic connections (Martin et al., 2000). Researchers have proposed various theories to interpret these changes. The most influential theory is Spike-Timing-Dependent Plasticity (STDP), which studies changes of synaptic connections as a function of presynaptic and postsynaptic neuronal activities (Hebb, 1949; Caporale and Dan, 2008). Nowadays, increasing studies indicate that in addition to neuronal activities, other factors can also modulate the changes of synaptic connections, such as neuromodulators and glia (Seol et al., 2007; Nadim and Bucher, 2014; Fremaux and Gerstner, 2015). Inspired by these findings, the concept of modulated STDP has gradually emerged based on the classic STDP (Fremaux and Gerstner, 2015). In our model, an energy-modulated STDP, which serves as a synaptic learning rule, is introduced to simulate the changes of synaptic weights (Yuan et al., 2018b), as follows:

(4) $$\begin{array}{r} \Delta w=f\left(\alpha \right)\cdot H\left(pre,\;post\right) \end{array}$$

where Δ*w* denotes the changes in synaptic weights at every time step, and *pre* and *post* denote the electrical activities of presynaptic and postsynaptic neurons, respectively. α is a dimensionless variable that measures the balance of energy metabolism in individual neurons. The function *f* (·) defines the regulation of the changes of synaptic weights by metabolic energy, and the function *H*(·) defines how the electrical activities of pre-synaptic and postsynaptic neurons determine the changes of synaptic weights.

The variable α is calculated as follows (Yuan et al., 2018b):

(5) $$\begin{array}{r} \alpha =\frac{\int_{t}^{t+\Delta T}E_{j}^{trans}d\tau }{\int_{t}^{t+\Delta T}E_{j}^{trans}d\tau +\int_{t}^{t+\Delta T}E_{j}^{integ}d\tau } \end{array}$$

(6) $$\begin{array}{r} E_{j}^{trans}=\overset{N_{j}^{trans}}{\underset{k=1}{\sum }}\left(E_{single}^{trans}\cdot w_{ij}\cdot \frac{\left(t-t_{k}\right)}{\tau_{trans}^{2}}\cdot e^{-\frac{\left(t-t_{k}\right)}{\tau_{trans}}}\right),\left(t\geq t_{k}\geq 0\right) \end{array}$$

(7) $$\begin{array}{r} E_{j}^{integ}=\overset{N_{j}^{integ}}{\underset{l=1}{\sum }}\left(E_{single}^{integ}\cdot \frac{\left(t-t_{l}\right)}{\tau_{integ}^{2}}\cdot e^{-\frac{\left(t-t_{l}\right)}{\tau_{integ}}}\right),\left(t\geq t_{l}\geq 0\right) \end{array}$$

where $E_{j}^{trans}$ and $E_{j}^{integ}$ are the metabolic energies consumed by synaptic transmission and dendritic integration of the *j-th* neuron at time *t*, respectively. $E_{single}^{trans}$ is the metabolic energy expended per action potential in synaptic transmission, and $E_{single}^{integ}$ is the metabolic energy expended per action potential in dendritic integration. $N_{j}^{trans}$ is the number of action potentials propagating to the *j-th* neuron before time *t*, and $N_{j}^{integ}$ is the number of action potentials generated by the *j-th* neuron before time *t*. τ_*trans*_ and τ_*integ*_ are the time constants of the changes in the energy consumption of synaptic transmission and dendritic integration, respectively. *t*_*k*_ is the time at which the *k-th* action potential reaches the *j-th* neuron, and *t*_*l*_ is the time at which the *l-th* action potential is generated by the *j-th* neuron. *w*_*ij*_ is the synaptic weight from the *i-th* neuron to the *j-th* neuron. It can be found from the above equations that α denotes the ratio of the metabolic energy consumed in synaptic transmission to the total metabolic energy consumed in synaptic transmission and dendritic integration within Δ*T*. The default values of the parameters in Equations (5–7) are Δ*T* = 12.5 s, τ_*trans*_ = 20 ms, τ_*integ*_ = 100 ms, $E_{single}^{trans}$ = 4.1 × 10^4^ ATPs, and $E_{single}^{integ}$ = 1.2 × 10^8^ ATPs.

The expressions of the functions *f* (·) and *H*(·) are as follows (Caporale and Dan, 2008; Yuan et al., 2018b):

(8) $$f\left(\alpha \right)=\frac{2}{1+e^{\lambda \cdot \frac{\Delta t}{\left|\Delta t\right|}\cdot \left(\alpha -c\right)}},\left(\lambda >0\right)$$

(9) $$H\left(pre,post\right)=\{\begin{array}{l} \xi {\left(1-w\right)}^{\upsilon }\cdot e^{-\frac{\left|\Delta t\right|}{\tau_{+}}},\;if\Delta t>0\\ -\xi \sigma w^{\upsilon }\cdot e^{-\frac{\left|\Delta t\right|}{\tau_{-}}},\;if\Delta t\leq 0 \end{array}$$

where λ is a dimensionless constant that specifies the extent to which metabolic energy affects neuronal electrical activities. ξ is the learning rate, and σ is an asymmetry parameter. τ_+_ and τ_−_ are the time constants of long-term potentiation and depression, respectively. Δ*t* = *t*_*post*_ – *t*_*pre*_ is equal to the latest spiking time of the postsynaptic neurons minus that of the presynaptic neurons. The parameter υ (0 ≤ υ ≤ 1) reflects the extent to which synaptic plasticity is affected by the current synaptic weight. A choice of υ = 0 leads to additive STDP, and a choice of υ = 1 leads to multiplicative STDP. Here, we chose υ = 1 to ensure that the network has better robustness. The value of the ratio α is equal to or infinitely close to the constant *c* when the metabolic energy consumed by synaptic transmission and dendritic integration are both at normal levels. According to experimental findings, the constant *c* is fixed at 0.75 (Howarth et al., 2012). When neurons reach energy balance, the energy-modulated STDP rule can be changed into the classical STDP learning rule. In this case, the increment or decrement in synaptic weights depends only on the electrical activities of the pre-synaptic and postsynaptic neurons. When the ratio α deviates from the constant *c*, the changes in synaptic weights can be regulated by metabolic energy, and the greater the deviation, the stronger the regulation. The default values of the parameters in Equations (8) and (9) are λ = 300, ξ = 0.001, υ = 1, σ = 1, and τ_+_ = τ_−_ = 10.0 ms.

### Evolution Rule

The evolution of neuronal networks in the brain endows humans a powerful ability to learn complex skills and adapt to changing environments. During the evolution, the elimination and formation of synaptic connections exist at the same time. The elimination of synaptic connections does not occur randomly but relies on neuronal electrical activity (Tessier and Broadie, 2009; Stoneham et al., 2010). Activity-dependent pruning mechanisms ensure that appropriate synaptic connections are conserved, while inappropriate ones are eliminated (Cody and Freeman, 2013). However, since the cellular and molecular mechanisms of activity-dependent synaptic pruning have not been fully revealed, it is somewhat difficult to determine directly which synaptic connections are appropriate or inappropriate. Nowadays, it is well-known that frequently used connections are generally conserved in general networks, while infrequently used connections are deleted (Navlakha et al., 2018). Thus, it is possible to indirectly determine whether synaptic connections are appropriate according to their usage frequency. Because of the synaptic learning rule mentioned above, the weights of frequently used synaptic connections can increase, while those of infrequently used synaptic connections can decrease. Therefore, the elimination of synaptic connections can be achieved as follows: during the evolution, synaptic weights throughout the neuronal network are sorted in ascending order, and the first *n* synaptic connections can then be eliminated at intervals.

Similar to the elimination of synaptic connections, their formation is also not random but exhibits some sort of preferential attachment and distance dependencies (Boccaletti et al., 2006; Ercsey-Ravasz et al., 2013). In many continuously growing real-world networks, a node's probability of receiving a new edge is proportional to the intensity of its activity, which is called preferential attachment by Barabasi and Albert (1999). This mechanism also exists in neuronal networks in the brain, i.e., the more active a neuron is, the easier it receives synaptic connections (Johnson et al., 2010). Preferential attachment may result in the emergence of hub neurons, which makes neuronal networks become scale-free (Johnson et al., 2009, 2010). It should be noted, however, that preferential attachment is not the only mechanism affecting the formation of synaptic connections. Otherwise, the neuron with the most synaptic connections would gradually connect to all other neurons in a neuronal network, which is obviously not in accord with experimental findings (Sporns and Betzel, 2016). Studies have further indicated that the connection probability between two neurons is related not only to the activity intensity of each neuron but also to the distance between them and decays exponentially with increased distance (Ercsey-Ravasz et al., 2013; Wang and Kennedy, 2016). For neurons with a large number of synaptic connections, the probability of forming synaptic connections between two faraway neurons is still very low, even if preferential attachment exists. Therefore, the distance-dependent connection probability *P*_*ij*_ from the *i-th* to *j-th* neurons in the neuronal network is first defined as follows:

(10) $$\begin{array}{r} P_{ij}=\frac{\mu {\left(\kappa \right)}^{2}\cdot \pi \left(\kappa_{i}\right)\cdot \pi \left(\kappa_{j}\right)}{{\left(d_{ij}/d_{min}\right)}^{o}},\\ i\neq j,\;i=1,2,\ldots ,N,\;j=1,2,\ldots ,N \end{array}$$

where κ is the average number of synaptic connections in the neuronal network, i.e., the actual synaptic density of the neuronal network. κ_*i*_ and κ_*j*_ are the number of synaptic connections incoming to the *i-th* and *j-th* neurons, respectively. *d*_*ij*_ is the arc distance between the *i-th* and *j-th* neurons along the spherical surface, as shown in Figure 1A. *d*_*min*_ is the minimum arc distance between neurons in the network, i.e., *d*_*min*_ = min{*d*_*ij*_}. *o* characterizes the influence of distance on the formation of synaptic connections between neurons. The function μ(·) defines the global probability that the average number of synaptic connections increases across the network. The function π(·) defines the local probability that the number of synaptic connections of a single neuron increases. In fact, the probability of the *i-th* neuron owning a new synaptic connection is defined as the product of the global probability μ(κ) and its local probability π(κ_*i*_) (Johnson et al., 2009, 2010; Millán et al., 2018a). Here, for simplicity, the formation of a new synaptic connection of neurons is assumed as an independent and identically distributed event. Therefore, the product of their respective probabilities owning a new synaptic connection, i.e., [μ(κ)·π(κ_*i*_)]·[μ(κ)·π(κ_*j*_)], is defined as the probability that the *i-th* and the *j-th* neurons are connected to each other. This is why the numerator of Equation (10) is μ(κ)^2^·π(κ_*i*_)·π(κ_*j*_). Based on previous studies on synaptic density during the development of neuronal networks in the brain (Johnson et al., 2009; Millán et al., 2018a), we have:

(11) $$\begin{array}{r} \mu \left(\kappa \right)=\frac{\eta }{N}\cdot \left(1-\frac{\kappa }{2\kappa_{\infty }}\right),\\ \pi \left(\kappa_{i}\right)=\frac{2\kappa_{i}}{\overset{N}{\underset{j=1}{\sum }}\kappa_{j}}-\frac{1}{N},\;i=1,2,\ldots ,N \end{array}$$

where η represents the total number of synaptic connections to be added or removed. The coefficient η indeed controls the descent rate of the synaptic density. κ_∞_ is the expected synaptic density of the neuronal network as the evolution time tends to ∞. Different values of κ_∞_ can reflect different changes in the descent magnitude of the synaptic density. According to the connection probability defined above, the formation of synaptic connections can be induced in the network in a biologically reasonable way. At intervals, the connection probabilities between any two neurons in the network are calculated, and then the first *m* pair of neurons in descending order of probability are connected to each other.

From the above evolution rule of the elimination and formation of synaptic connections, it can be found that *n* synaptic connections are eliminated and *m* synaptic connections are formed at intervals. According to the global probability μ(·), the values of *n* and *m* can be calculated as follows:

(12) $$\begin{array}{r} n=\left(1-\mu \left(\kappa \right)\right)\cdot N,\\ m=\mu \left(\kappa \right)\cdot N \end{array}$$

Here, the number of eliminated synaptic connections equals that of newly formed synaptic connections when μ(κ) = 0.5. This means that the synaptic density of the neuronal network becomes stable, i.e., κ = κ_∞_.

The proposed model is implemented in MATLAB, in which the GPU is adopted for parallel operations; the code is available upon request. For the sake of simplicity, the default values of some parameters in the model are listed here: *N* = 1,250, *M* = 500, η = 1,000, κ_∞_ = 300, *o* = 0.8, *time step* = 0.25 ms. The number of synaptic connections in the network is ~1.5 million. During the simulation, the parameters are set according to the above values unless otherwise specified. In addition, initial values of all connections (*w*_*ij*_, *b*_*kj*_) in the network are generated randomly according to a uniform distribution.

In the numerical simulation of the above model, the change of network structure, the calculation of energy metabolism, and the updating of synaptic weights and neuron membrane potentials are involved. It should be noted that the time scales of these processes are different. In the numerical simulation, the synaptic weights and neuron membrane potentials are updated at each time step. Because the energies consumed by synaptic transmission and dendrite integration of neurons are calculated by counting the total number of spikes within a period of time. If the time period is too short, the calculated result is not accurate enough and changes sharply. Therefore, at each time step, the average energy consumption of neurons is calculated according to the total number of spikes in the past period time Δ*T* = 12.5 s (50,000 time steps). As for the change of the network structure, its time scale is related to the parameters of the synaptic learning rule. After the network structure changes, the synaptic weights will be adjusted automatically according to the synaptic learning rule. When the synaptic weights tend to be stable, the next change of the network structure begins. In our numerical simulation, the formation and the elimination of synaptic connections are performed every ~50,000 time steps.

## Results

### Network Connectivity

**Figure 2:**
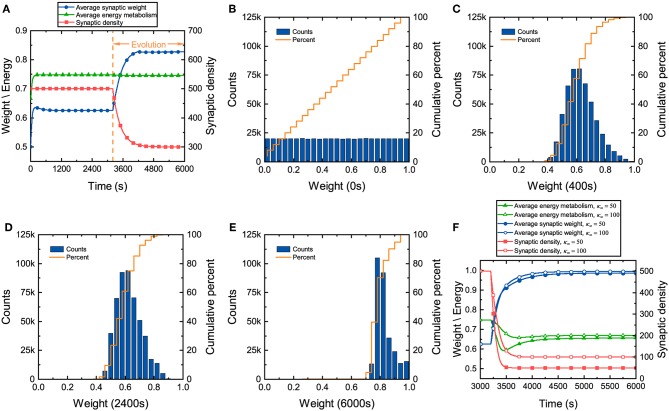
Changes in several characteristics of the neuronal network during evolution. **(A)** Changes in the average synaptic weight, synaptic density, and average energy metabolism. **(B–E)** The distributions and cumulative frequency curves of the initial synaptic weights at 0, 400, 2,400, and 6,000 s. **(F)** Changes in synaptic density, average synaptic weight, and average energy metabolism when the parameter κ_∞_ is set to 50 and 100.

During the development of neuronal networks, synapses are overproduced and then pruned back over time, whereas in engineered networks, connections are initially sparse and are then added over time (Navlakha et al., 2015, 2018). The differences in the construction strategy make neuronal networks exhibit some unique characteristics during the development. Synaptic weights in the neuronal network, which directly reflect the tightness of network connectivity, along with synaptic density and energy metabolism, are investigated here. We assume that continuously generated external signals obey a normal distribution *N*(40, 10), and neurons in the network receive these signals at random. It can be found from Figure 2A that before network evolution, the average synaptic weight still changes due to the synaptic learning rule (Yuan et al., 2018b). The average synaptic weight is initially ~0.5 and then quickly increases to ~0.64 in a short time and eventually tends to stabilize. The distributions of the synaptic weights at different moments are also drawn in Figures 2B–E. Initial values of the synaptic weights are assigned randomly according to a uniform distribution, as shown in Figure 2B. As the average synaptic weight stabilizes, the synaptic weights exhibit an approximately normal distribution, as shown in Figure 2C. Comparing Figure 2B with Figure 2C, it is obvious that the synaptic weights change dramatically in the initial stage and quickly concentrate around ~0.6 in a very short time. After long-term stabilization of the average synaptic weight, its distribution is shown in Figure 2D. Obviously, compared with Figure 2C, the distribution of the synaptic weights further deviates from a normal distribution. This suggests that there are still some slight differences in their distribution. Even though the average synaptic weight stabilizes, the synaptic weights continue to regulate themselves slightly.

In the synaptic learning rule mentioned above, the energy metabolism of neurons is measured by a dimensionless variable α in Equation (4). In our numerical simulation, the energy metabolism of the entire network is characterized by the average energy metabolism of all neurons. Initially, the average energy metabolism has a similar trend as the average synaptic weight. It can then be inferred that the dramatic change in the synaptic weights makes the average energy metabolism quickly reach the biologically reasonable energy level regulated by the synaptic learning rule. When the average energy metabolism becomes stable, the slight change in the synaptic weights should be derived from the interactions between neurons in the network.

Once the distribution of the synaptic weights becomes substantially stable, the network starts evolving according to the given evolution rule. From Figure 2A, it can also be found that although the formation and elimination of synaptic connections can occur simultaneously during evolution, the synaptic density of the network still decreases, which is similar to neuronal networks in the brain (Goyal and Raichle, 2013; Navlakha et al., 2018). Furthermore, the average synaptic weight increases gradually with the decrease of the synaptic density. As the average synaptic weight tends to stability, the distribution of the synaptic weights is shown as Figure 2E. Compared with the distribution of the synaptic weights before evolution, the synaptic weights increase significantly after evolution, showing an irregular distribution. Although the average synaptic weight and synaptic density change dramatically during evolution, the average energy metabolism remains unchanged. Therefore, it is necessary to further study whether the average energy metabolism remains unchanged during the decline of synaptic density. Figure 2F shows that the average synaptic weight increases as the synaptic density decreases, but the average energy metabolism is not always stable. When the synaptic density drops to a very low level, the average energy metabolism begins to decline. The lower the synaptic density, the lower the average energy metabolism, and the average synaptic weight tends to the upper limit set by the network model.

It is well-known that synaptic transmission and dendritic integration are two main metabolically expensive processes in information processing in neuronal networks (Howarth et al., 2012; Yuan et al., 2018a). During development, the connectivity of neuronal networks must ensure that the energy metabolism of each neuron is at a normal level. In our proposed network, the synaptic weights and the synaptic density, which describe the connectivity from different perspectives, determine the amount of information received by neurons, thus affecting the synaptic transmission and dendritic integration. It can be concluded from the above simulation results that although neuronal network construction is determined by the genes of the organism (Thompson et al., 2001; Glahn et al., 2010), it is also affected by energy metabolism in reality. The synaptic weights and the synaptic density counterbalance each other so that the energy metabolism of each neuron is at a normal level. In addition, the synaptic weights increase during evolution as the synaptic density decreases, suggesting that the efficiency of frequently used synaptic connections in the network has been improved. Note that the range of changes for the synaptic weights is limited. If the synaptic density of the neuronal network is very sparse, failure of the energy metabolism can also occur in some neurons even if all the synaptic weights become very large.

### Network Efficiency

As the structural basis of human cognitive function, neuronal networks in the brain continuously receive a variety of sensory information and use it to make decisions (Avena-Koenigsberger et al., 2018). This means that neuronal networks during development should be optimized enough to adapt to such heavy information processing (Laughlin and Sejnowski, 2003; Bullmore and Sporns, 2012). At this stage, the synaptic density of neuronal networks can suffer from significant decline. This naturally raises the questions of whether network efficiency is related to synaptic density and what the relationship between them is. In this section, we propose a criterion for evaluating network efficiency and then study their relationship.

For neuronal networks in the brain, in addition to the energy consumed by signaling activities such as synaptic transmission and dendritic integration, metabolic energy is also required to maintain the basic housekeeping tasks within synapses (Howarth et al., 2012). Under the conditions that the normal functions of neuronal networks are maintained well and that their total energy supply is constant, lower synaptic density means that more energy can be used for signaling activities. Then, reducing the synaptic density contributes to improving the network efficiency. However, their average shortest path length increases as the synaptic density of neuronal networks decreases, thereby resulting in longer signal transmission times. Therefore, the network efficiency worsens if the synaptic density is reduced excessively. To improve the network efficiency, there should be a trade-off between the average shortest path length and the synaptic density of neuronal networks. Based on the discussions above, for simplicity, the reciprocal of the product of the average shortest path length and the synaptic density of neuronal networks is used to evaluate the network efficiency. The larger the value of the reciprocal, the higher the network efficiency.

**Figure 3:**
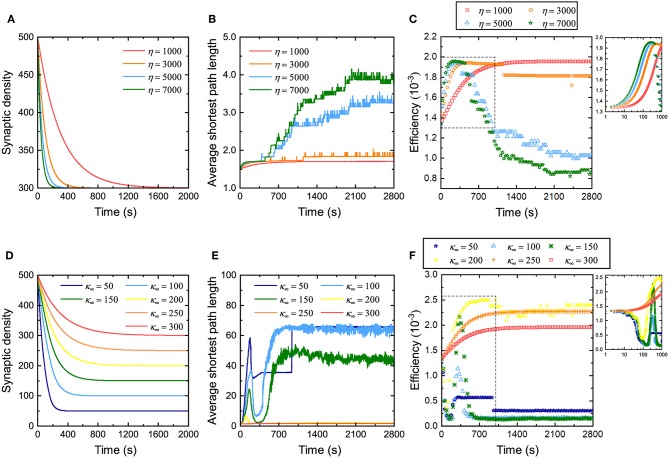
Changes in the network efficiency under different evolutionary conditions. **(A–C)** Changes in synaptic density, average shortest path length, and network efficiency of the neuronal network with different coefficients η. The network evolution is simulated four times. In each simulation, all parameters remain unchanged except for the coefficient η. Inset in **(C)** shows a partial enlargement of the data from the dashed box. **(D–F)** Changes in synaptic density, average shortest path length, and network efficiency of the neuronal network with different expected synaptic densities κ_∞_. The network evolution is simulated six times. In each simulation, all parameters remain unchanged except for the expected synaptic density κ_∞_. The inset in **(F)** shows a partial enlargement of the data from the dashed box. Note that the data in **(C,F)** are sampled every 30 s. Since the network efficiency changes rapidly in the first 1,000 s, for the sake of observation, the data sampled every second in the initial 1,000 s are drawn in the insets in the upper right corner.

Some studies have discussed the relationship between network efficiency and synaptic density (Navlakha et al., 2015, 2018), but they do not specify how the rate and magnitude of the decrease in synaptic density affects network efficiency. Therefore, the influence of the rate of decrease of the synaptic density on network efficiency during evolution is first investigated. From Figure 3A, it can be found that for neuronal networks with different coefficients η, their synaptic density decreases from the same initial value to the expected value at different descent rates. The larger the coefficient η, the faster the synaptic density decreases to the expected value. Similarly, their average shortest path length gradually increases from the same initial value to different values in fluctuation, as shown in Figure 3B. The larger the coefficient η is, the more the average shortest path length increases, and the more drastic its fluctuation will be. The network efficiency corresponding to different coefficients η is shown in Figure 3C. Obviously, in the first stage, the neuronal networks have a similar efficiency. As the network evolves, the network efficiency exhibits different trends with different coefficients η. The larger the coefficient η, the faster the network efficiency reaches the maximum. The maximum network efficiency is almost identical regardless of how fast the synaptic density decreases. It is worth noting, however, that after the network efficiency reaches its maximum, it may not be stable. In particular, for a neuronal network with a very large coefficient η, after its efficiency reaches the maximum, it falls to a lower level than before the evolution. According to the definition, the larger the coefficient η, the more synaptic connections are eliminated and formed at intervals. For these neuronal networks, it is difficult to maintain stability when the network efficiency reaches its maximum because too many synaptic connections are eliminated or formed at intervals, resulting in drastic changes in the structure of the neuronal network.

Subsequently, we also investigated the influence of the amplitude of the decrease in synaptic density on network efficiency. Figure 3D shows that the synaptic densities of the neuronal networks decrease from the same initial value to different expected values κ_∞_. Although the coefficient η retains the same in these simulations, the descent rates are still different due to different descent magnitudes. Clearly, the larger the descent magnitude, the shorter the time it takes for the synaptic density to fall to the expected level. Similarly, for neuronal networks with different expected synaptic densities κ_∞_, their average shortest path length gradually increases from the same initial value to different values in fluctuation, as shown in Figure 3E. The larger the expected synaptic density κ_∞_, the more the average shortest path length increases, and the more intense its fluctuation. The network efficiencies corresponding to these expected synaptic densities are shown in Figure 3F. It can be found that the network efficiency gradually increases and stabilizes at a higher level only when the descent magnitude is small. Otherwise, the network efficiency fluctuates dramatically and eventually falls to a lower level. For example, when the synaptic density decreases to 300 (i.e., a 20% drop), the network efficiency eventually increases to 2.0 × 10^−3^; however, when the synaptic density decreases to 100 (i.e., an 80% drop), the network efficiency eventually decreases to 1.25 × 10^−4^.

The relationship between network efficiency and synaptic density has been discussed based on the criterion for evaluating network efficiency above. The simulation experiments here validate our analyses again. Although reducing the synaptic density is beneficial to improving the network efficiency, excessively reducing it leads to a sharp increase in the average shortest path length, as shown in Figure 3E, thereby offsetting the increase in the network efficiency caused by decreasing the synaptic density. This is why the curves in Figure 3F show very different trends. This also suggests that the synaptic density must only be reduced within a certain range to improve the network efficiency.

The above experiments study the relationship between synaptic density and network efficiency by changing the rate and amplitude of the decrease in synaptic density. Two conclusions can be drawn from Figure 3. First, when the descent rate of the synaptic density changes but its descent amplitude remains unchanged, network efficiency can always increase to almost the same maximum level. The difference lies in whether the maximum network efficiency is stable. The larger the descent rate of the synaptic density, the more difficult it is for the network efficiency to be stable at the maximum level. Thus, the descent rate of the synaptic density mainly determines whether the network efficiency remains stable at this maximum level. Second, when the descent amplitude of the synaptic density changes but its descent rate remains unchanged, conversely, the network efficiency can show very different trends and eventually stabilize at different levels. Reducing the synaptic density appropriately can improve network efficiency, while excessively reducing the synaptic density can degrade network efficiency. Therefore, the descent amplitude of the synaptic density mainly determines the maximum level of the network efficiency.

### Network Degree Distribution

During the development of neuronal networks in the brain, some synaptic connections are still formed, although the synaptic density decreases as a whole. In our network model, both preferential attachment, a ubiquitous mechanism for network construction (Barabasi and Albert, 1999; Johnson et al., 2010), and the distance between neurons play critical roles in the formation of synaptic connections during evolution. In this section, we study the roles of these two factors in evolution by analyzing the degree distribution of the neuronal network. Here, the degree can be subdivided into the indegree and outdegree. The former denotes the number of synaptic connections incoming to a neuron, while the latter denotes the number of synaptic connections outgoing from a neuron.

**Figure 4:**
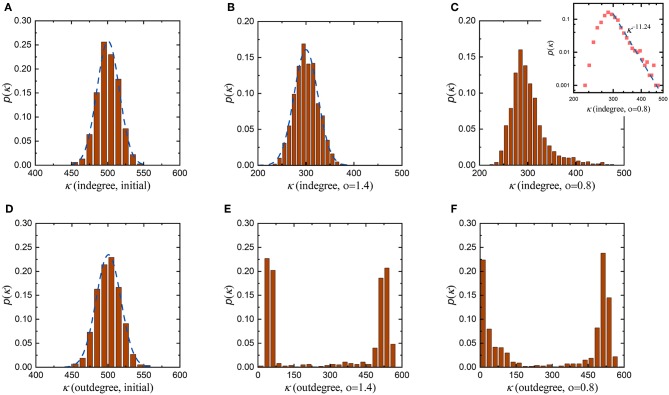
Changes in the degree distributions of the neuronal network before and after evolution. To study the roles of the distance between neurons, the evolution of the neuronal network with the coefficient *o* set to different values is simulated. In each simulation, all parameters remain unchanged except for the coefficient *o*. **(A)** The initial indegree distribution of the neuronal network before evolution. **(B,C)** The indegree distributions of the evolved neuronal network when the coefficient *o* is set to 1.4 and 0.8, respectively. **(D)** The initial outdegree distribution of the neuronal network before evolution. **(E,F)** The outdegree distributions of the evolved neuronal network when the coefficient *o* is set to 1.4 and 0.8, respectively.

According to the proposed evolution rule above, the coefficient *o* in Equation (10) characterizes the influence of the distance between neurons on the formation of synaptic connections. Since synaptic connections in the neuronal network are formed randomly before evolution, the initial indegree and outdegree distributions are very similar, both of which obey a normal distribution with an average value of 500, as shown in Figures 4A,D. This suggests that before evolution, neurons in the neuronal network receive signals from, on average, 500 neurons while also sending signals to, on average, 500 neurons. Then, we set the coefficient *o* to 1.4 to allow the neuronal network to evolve. As the average synaptic weight, the average energy metabolism and the synaptic density of the neuronal network stabilize, its indegree and outdegree distributions change as shown in Figures 4B,E, respectively. After evolution, the indegree and outdegree of the neuronal network exhibit obviously different distributions. Compared with the initial indegree distribution, the indegree distribution of the evolved neuronal network still obeys a normal distribution, and the biggest change is that the average of this distribution decreases from 500 to 300. The outdegree distribution of the neuronal network after evolution, however, changes dramatically, from a normal distribution to a bimodal distribution. This means that, after evolution, the average number of synaptic connections incoming to a single neuron in the neuronal network has only decreased from 500 to 300, while the number of synaptic connections outgoing from a single neuron shows significant polarization. On the whole, some neurons in the evolved neuronal network transmit signals to fewer than 100 neurons, while others transmit signals to at least 500 neurons. Subsequently, we set the coefficient *o* to 0.8 and repeat the evolution. Similarly, as the average synaptic weight, the average energy metabolism and the synaptic density stabilize, the indegree and outdegree distributions change as shown in Figures 4C,F, respectively. The indegree and outdegree distributions of the evolved neuronal network are still very obviously different. The outdegree distribution again changes from a normal distribution to a bimodal distribution. However, the indegree distribution does not obey a normal distribution, as with the greater value of the coefficient *o* mentioned above, but instead follows a typical power law distribution. Figure 4C shows that most neurons in the evolved neuronal network receive signals from 250 to 350 synaptic connections, but a small number of neurons receive signals from more than 400 synaptic connections. This means that such a small number of neurons may gradually evolve into hub neurons when the coefficient *o* is set to 0.8, as shown in Figure S1. In fact, previous studies have shown that neuronal networks in certain brain regions are scale-free and have hub neurons, which may coordinate network activities (Bonifazi et al., 2009; van den Heuvel and Sporns, 2013).

To comprehensively investigate the roles of the distance between neurons in network evolution, more simulations are carried out with various values of the coefficient *o*, as shown in Figures S2, S3. Some conclusions can be drawn from Figure 4 and Figures S2, S3. First, regardless of the value of the coefficient *o*, the outdegree distribution of the evolved neuronal network changes from a normal distribution to a bimodal distribution. Second, when the value of the coefficient *o* is large, the indegree distribution of the evolved neuronal network still obeys a normal distribution; however, when the value of the coefficient *o* is small, its indegree distribution gradually changes from a normal distribution to a power law distribution. Third, when the value of the coefficient *o* is sufficiently small, some hub neurons can be connected to almost all the neurons in the network during evolution. According to the evolution rule, the larger the coefficient *o*, the greater the influence of the distance between neurons on the formation of synaptic connections; otherwise, the influence will be smaller. Combining the simulation results with the meaning of the coefficient *o*, it can be further concluded that the formation of hub neurons is closely related to the distance between neurons. If distance plays a weaker role in the evolution of the neuronal network, then neurons are connected not only to nearby neurons but also to distant neurons; otherwise, neurons are only connected to nearby neurons. In other words, the distance between neurons determines whether hub neurons exist and whether the indegree distribution of the evolved neuronal network is normally distributed or power-law distributed.

Another question that needs to be addressed is why the indegree and outdegree of the evolved neuronal network show very different distributions. According to the evolution rule, preferential attachment is used to induce synaptic connections during evolution. The essence of this strategy is that the more active a neuron is, the more synaptic connections it has. There is no doubt that the more signals a neuron receives, the more active it becomes. The total number of signals received by neurons depends mainly on the number of afferent synaptic connections they have made. In this paper, the activity of neurons is only evaluated by the number of afferent synaptic connections, i.e., the indegree of the neurons. Obviously, the outdegree of the neurons does not directly affect their ability to form synaptic connections. Therefore, the indegree and outdegree of the evolved neuronal network show very different distributions.

**Figure 5:**
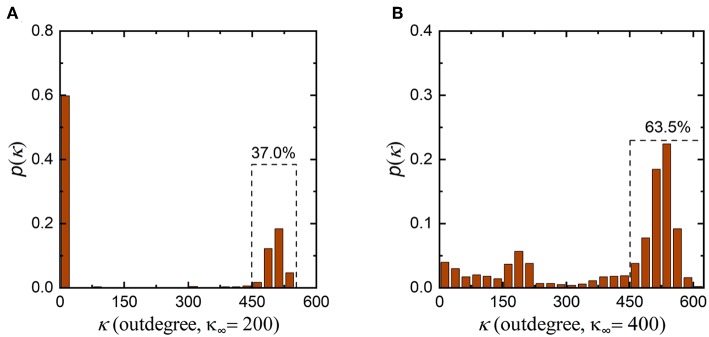
Changes in the outdegree distribution of the network after the evolution. **(A)** The outdegree distribution of the evolved neuronal network when the expected synaptic density is set to 200. The number of neurons with a larger outdegree is obviously less than the number with a smaller outdegree. **(B)** The outdegree distribution of the evolved network when the expected synaptic density is set to 400. The number of neurons with a larger outdegree is obviously greater than the number with a smaller outdegree.

In addition, it is worth noting that the synaptic density of the neuronal network decreases from 500 to 300 during evolution, indicating that the number of synaptic connections in the neuronal network decreases. According to graph theory, in this case, the average indegree and outdegree of the neuronal network should decrease simultaneously with the same magnitude. From Figure 4 and Figures S1, S2, during evolution, the indegree of neurons in the neuronal network decreases as expected, but the outdegree of some neurons in the network does not obviously change and remains at ~500. To ensure that the average indegree and outdegree of the neuronal network are equal, the outdegree of some neurons in the neuronal network should be greatly decreased. This is why the outdegree of the neuronal network shows a bimodal distribution. According to the interpretation above, if the expected synaptic density of the neuronal network is set to a lower value, it can be inferred that the number of neurons with a smaller outdegree is obviously greater than the number of neurons with a larger outdegree after evolution. In contrast, a higher expected synaptic density means that the number of neurons with a smaller outdegree is apparently less than the number of neurons with a larger outdegree after evolution. To verify the rationality of this inference, we set the expected synaptic density of the neuronal network to different values and repeat the simulation, as shown in Figure 5. It is obvious that the results are consistent with our inference. If the expected synaptic density is set to 200, the number of neurons with a larger outdegree accounts for 37% of the total number of neurons in the evolved network. If the expected synaptic density is set to 400, the number of neurons with a larger outdegree accounts for 63.5% of the total number of neurons in the evolved network.

## Discussion and Conclusion

During the development of the brain, the synaptic density of neuronal networks increases rapidly and then gradually decreases to a stable level (Navlakha et al., 2018). The study described in this paper aims to investigate changes in the structure of neuronal networks during brain development. Our proposed model imitates the gradual decrease of the synaptic density to a stable level. Although the synaptic density decreases gradually at this stage, the formation of synaptic connections still coexists with the elimination of synaptic connections. In this model, a mechanism called preferential attachment is used to induce the formation of synaptic connections, while synaptic connections are eliminated according to synaptic weights obeying the synaptic learning rule (Caporale and Dan, 2008) and the reward regulation mechanism (Fremaux and Gerstner, 2015; Yuan et al., 2018b). Using this model, our research focuses mainly on three aspects: connectivity, efficiency, and degree distribution.

Through the simulation results above, we find that the synaptic weight and the synaptic density, two aspects of the connectivity of the neuronal network, determine the energy consumption of the neuronal network in information processing. When the energy metabolism level in the neuronal network is fixed, the synaptic weight, and the synaptic density counterbalance to each other; that is, one side increases, and the other side inevitably decreases. It can be further inferred that the connectivity of the neuronal network can be regulated by energy metabolism to some extent during evolution. Although genes determine the strategy of neuronal network construction, energy metabolism also plays an important role in the implementation of this strategy (French and Pavlidis, 2011). There are several points worth discussing about this conclusion. For example, energy metabolism is affected by many factors, such as body temperature, food intake, mental state, and sleep (Dworak et al., 2010; Yu et al., 2012), which means that these factors also indirectly affect neuronal network construction during development. Many studies have shown that changes in body temperature can affect metabolic rate and indirectly affect the development of neuronal networks in the brain (White et al., 2006). Warm temperatures can promote various biochemical processes and facilitate energy-efficient cortical action potentials (Yu et al., 2012). Therefore, it can be inferred that the connectivity of neuronal networks may be indirectly regulated by body temperature via energy metabolism in the brain. Furthermore, energy metabolism in the brain is also closely related to food intake, mental state and sleep (Dworak et al., 2010). The structure of neuronal networks is disrupted if food is scarce, one's mental state is poor, and/or sleep is inadequate during brain development.

Our second task is to study the relationship between synaptic density and network efficiency by varying the descent rate and amplitude of the synaptic density. We find that the descent rate of the synaptic density mainly determines whether the neuronal network can remain stable at maximum efficiency, while the descent magnitude of the synaptic density mainly determines the maximum efficiency of the neuronal network. Our simulation results show that the larger the descent rate, the more difficult it is for the network efficiency to remain stable at the maximum level. In addition, a too small or too large decrease in synaptic density can lead to a decrease in maximum network efficiency. In our simulation results, a reduction of ~50% in the synaptic density can maintain the efficiency of the neuronal network at a high level, as shown in Figure 3D. Many previous experiments have shown that human synaptic density peaks around age 2 and then decreases by 50 ~ 60% in adulthood (Navlakha et al., 2015, 2018). Obviously, our simulation results are consistent with the results of biological experiments, which can partly explain why the synaptic density of neuronal networks is reduced by nearly half during brain development.

Our third task is to study the influence of the distance between neurons on the formation of synaptic connections during evolution by analyzing the degree distribution of the neuronal network. We find that during evolution, the distance between neurons has a very significant influence on the formation of synaptic connections, thereby leading to obvious changes in the indegree and outdegree distributions of the neuronal network. If the influence of the distance is large, the indegree distribution of the evolved neuronal network always obeys a normal distribution. If the influence of the distance decreases, the indegree distribution gradually shifts from a normal distribution to a power law distribution after the evolution. For the outdegree, regardless of whether the influence of the distance is strong or weak, its distribution is always bimodal. In fact, a power law distribution is the most basic characteristic of scale-free networks (Barabasi and Albert, 1999). Our simulation results show that the evolved neuronal network has the characteristics of a scale-free network to a certain extent, which has also been confirmed by previous studies (Johnson et al., 2009, 2010; Pritchard et al., 2014). In addition, our simulation results further show that the scale-free characteristic of the neuronal network is affected by its spatial distribution. The sparser the spatial distribution of the neuronal network is, the larger the distance between neurons is, and the greater the influence they have on the formation of synaptic connections. In this case, the indegree distribution of the neuronal network is more biased toward a normal distribution. Conversely, a denser spatial distribution of the neuronal network indicates that the indegree distribution of the neuronal network will tends toward a power law distribution. In fact, previous studies have already discussed the influence of the distance between nodes on general scale-free networks (Mukherjee and Manna, 2006). Only in networks where the average distance between nodes is relatively close can nodes be connected not only to nearby nodes but also to distant nodes, thus showing the scale-free characteristic. Clearly, our conclusions are consistent with previous studies.

Recently, a brain developing model, which combines an auto-associative neuronal network with an evolving mechanism for the birth and death of synapses, is proposed to study the relationship between structural and functional properties of neuronal networks (Millán et al., 2018a,b, 2019). Millán's model is similar to ours and also involves network dynamics and generation rules. In addition to similar neuronal dynamics and synaptic learning rules, preferential attachment is also employed to achieve the formation of new synaptic connections between neurons. Nevertheless, there are some significant differences between Millán's model and ours. In Millán's model, synaptic connections are eliminated based on probabilities, which are calculated by the synaptic current and the synaptic density. In our model, synaptic connections are eliminated according to synaptic weights. Furthermore, the network generation rules in our model contain some factors that reflect the spatial distance between neurons, which is not considered in Millán's model. The numerical simulation results of Millán's model demonstrate that the appearance of hubs and heterogeneity can greatly improve the stability of the memory patterns (Millán et al., 2019), and the existence of synaptic pruning is critical in providing ordered stationary states and stable memories (Millán et al., 2018a,b). Whereas, our numerical simulation exhibits how the network connectivity and the network efficiency changes during the decline of the synaptic density, and how the spatial distance between neurons affects the appearance of hubs and heterogeneity. Note that, because our model uses a different biologically reasonable strategy to eliminate synaptic connections, the degree of neurons does not show a subcritical distribution similar to that of Millán's model.

There is another point worth discussing about the strategy of synaptic growth and pruning in our model. As we known, advances in technology have led to a growing understanding for the microscopic mechanisms of synaptic growth and pruning (Navlakha et al., 2018). However, there are too many factors affecting synaptic growth and pruning, so it is difficult to quantify and integrate all factors into a neuronal network model. The interaction of these factors makes synaptic growth and pruning show some mesoscale characteristics, such as distance dependence and power law distribution. It is a potential way to introduce the mesoscale characteristics into neuronal network models without caring about the microscopic mechanisms. Nowadays, a variety of neuronal network models are proposed, such as Convolution Neural Network, Spike Neural Network, and Recurrent Neural Network (Shen et al., 2017; Xu et al., 2018). Although these models make remarkable achievements in specific tasks, the design of their structures does not exhibit the characteristics of distance dependence, power law distribution. Therefore, the distance-dependent synaptic growth and weight-based synaptic pruning in our model may provide new ideas for the design of neuronal network models.

## Data Availability

The datasets generated for this study are available on request to the corresponding author.

## Author Contributions

YY and TF conceived the project and designed the experiments. All authors contributed to the development of the concepts presented in the paper. YY performed the experiments and data analysis. All authors helped write the manuscript.

### Conflict of Interest Statement

The authors declare that the research was conducted in the absence of any commercial or financial relationships that could be construed as a potential conflict of interest.
