# Supplementary material for: Structural Insights Into the Dynamic Evolution of Neuronal Networks as Synaptic Density Decreases
Source: Front Neurosci. 2019 Aug 22;13:892. doi: 10.3389/fnins.2019.00892 (PMC6714520; doi:10.3389/fnins.2019.00892)
Supplement: Supplementary file 1 [file Data_Sheet_1.docx]

## Supplementary Materials

**Figure S1.**


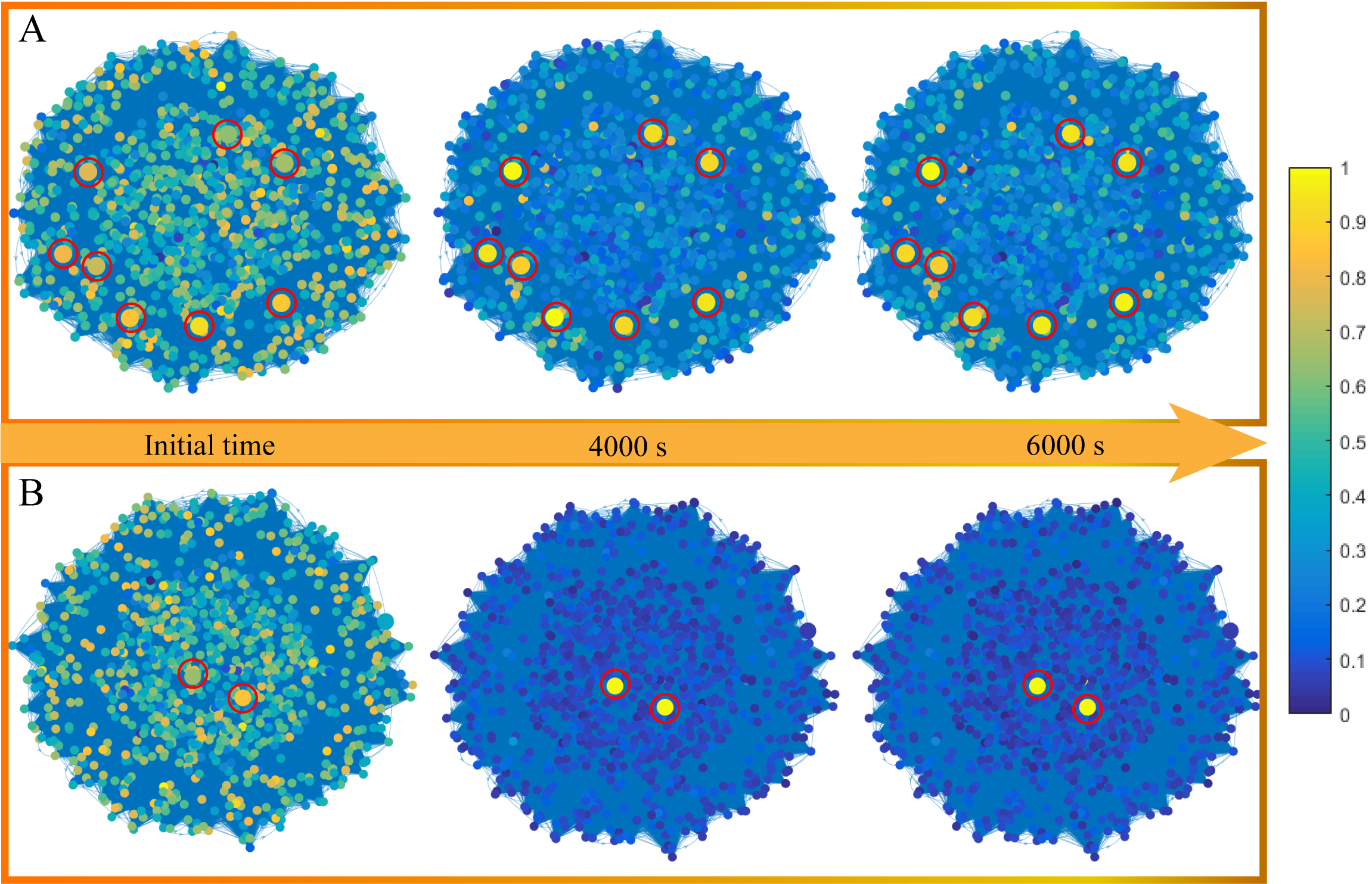


**Figure S1 | The structural evolution of the neuronal network and hub formation with the coefficient *o* set to different values.** The indegrees of all neurons in the network are normalized, and their values are characterized by different colours. The larger the indegree is, the closer the neuronal colour is to yellow. In contrast, the smaller the indegree is, the closer the neuronal colour is to blue. Hub neurons in the network are marked by red circles. **(A)** The evolution of the network when the coefficient *o* is set to 0.8. Initially, the difference in the indegree of the neurons in the network is small. Subsequently, the indegree of most neurons gradually decreases, but the indegree of a few neurons remains almost unchanged. **(B)** The evolution of the neuronal network when the coefficient *o* is set to 0.6. Initially, the difference in the indegree of neurons in the network is small. Subsequently, the indegree of most neurons gradually decreases, but the indegree of two of the neurons increases.

**Figure S2.**


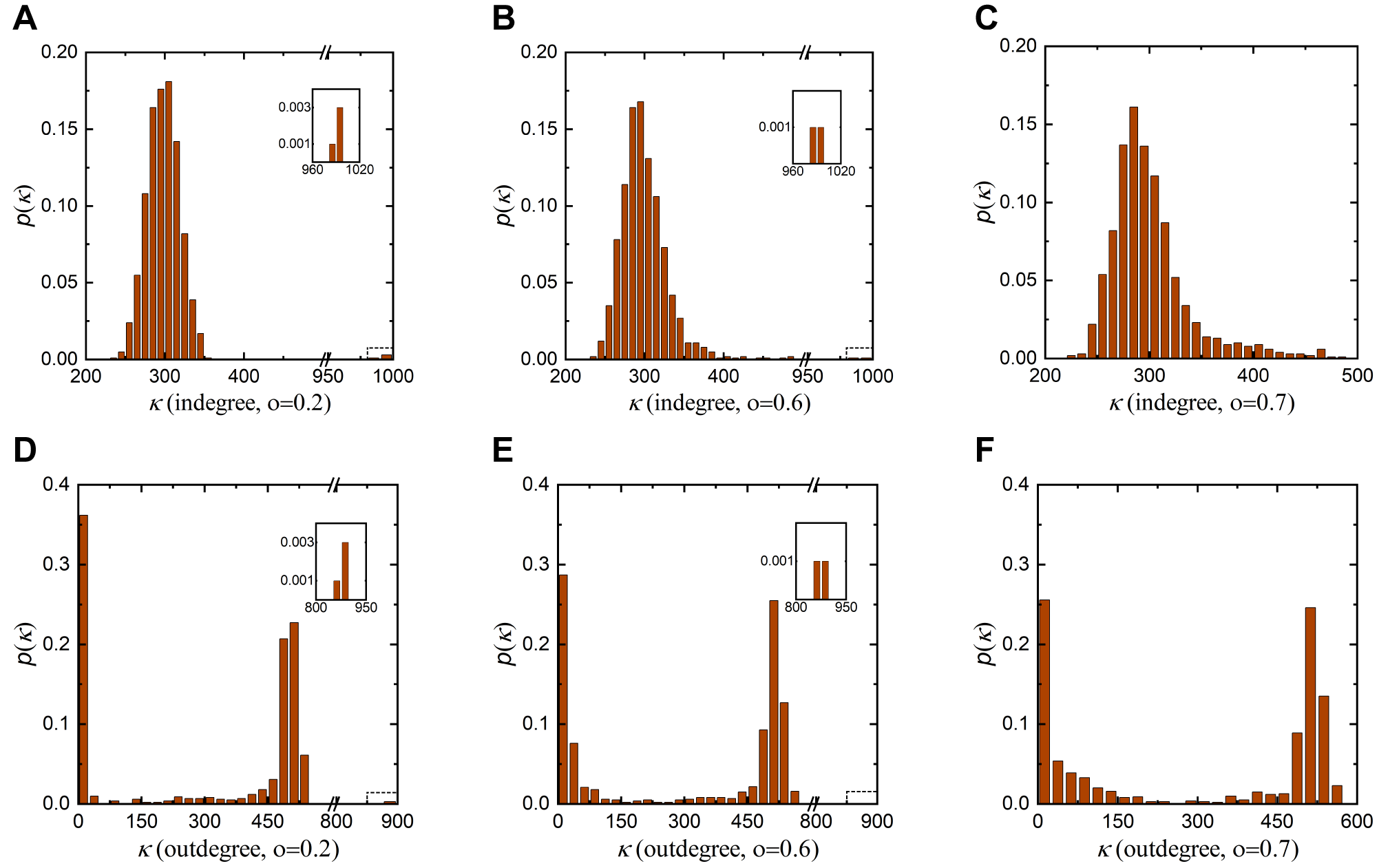


**Figure S2 | Changes in the degree distributions of the neuronal netwo­­rk before and after evolution.** **(A, B, C)** The indegree distributions of the evolved network when the coefficient *o* is set to 0.2, 0.6, and 0.7, respectively. **(D, E, F)** The outdegree distributions of the evolved network when the coefficient *o* is set to 0.2, 0.6, and 0.7, respectively. Note that the insets illustration in the upper right corner of **(A)**, **(B)**, **(D)**, and **(E)** are enlargements of the respective dotted boxes in the lower right corners of the panels.

**Figure S3.**


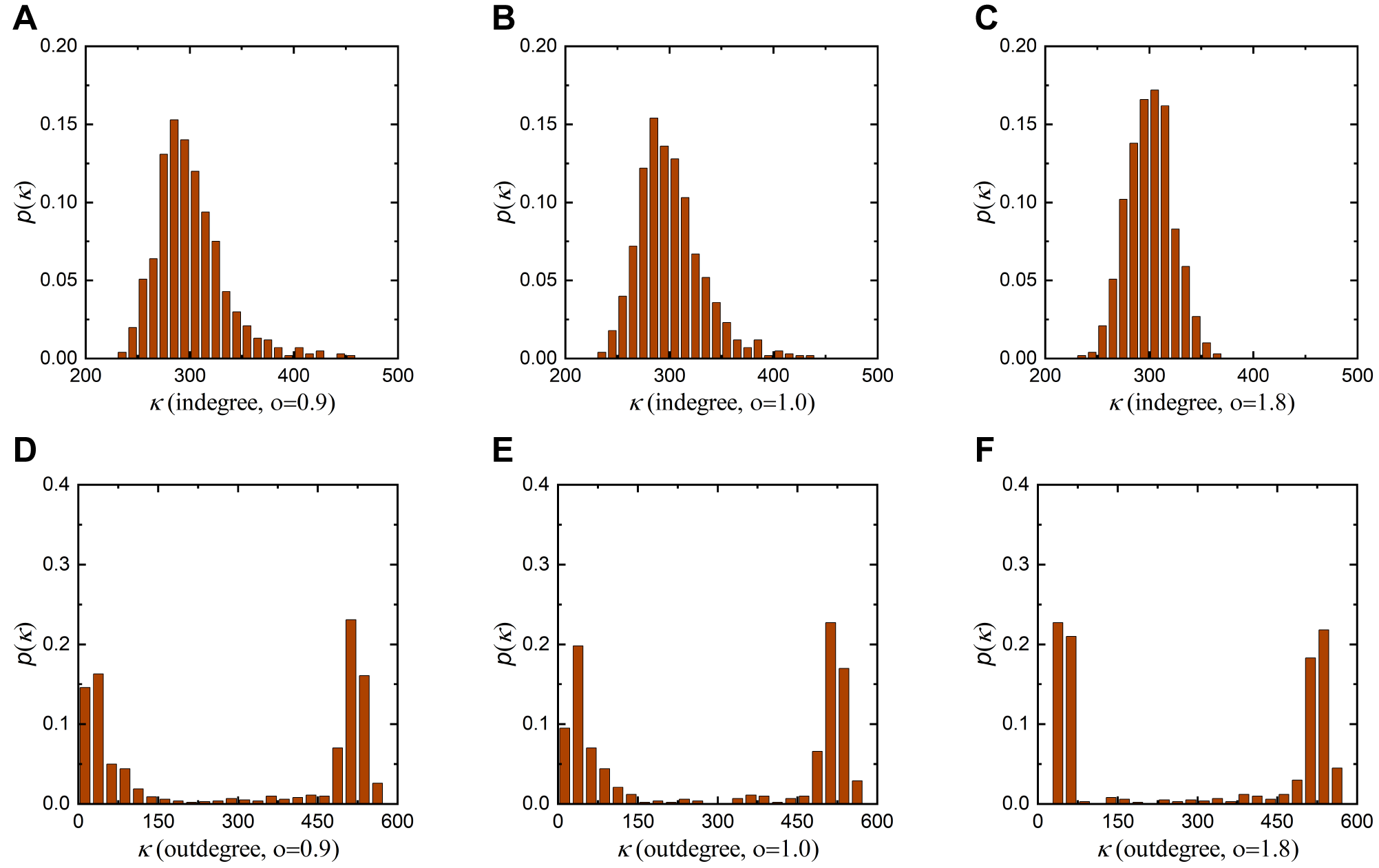


**Figure S3 | Changes in the degree distributions of the neuronal netwo­­rk before and after evolution.** **(A, B, C)** The indegree distributions of the evolved network when the coefficient *o* is set to 0.9, 1.0, and 1.8, respectively. **(D, E, F)** The outdegree distributions of the evolved network when the coefficient *o* is set to 0.9, 1.0, and 1.8, respectively.
